# Supplementary material for: Economic evaluation of Wolbachia deployment in Colombia: A modeling study
Source: PLoS One. 2025 Apr 30;20(4):e0307045. doi: 10.1371/journal.pone.0307045 (PMC12043165; doi:10.1371/journal.pone.0307045)
Supplement: S1 Table — (PDF) [file pone.0307045.s001.pdf]

Supporting Information S1 Table

Input data for target cities

For

Economic evaluation of *Wolbachia* deployment in Colombia: A modeling study

Plos One, 2025. https://doi.org/10.1371/journal.pone.0307045

By

Donald S. Shepard, PhD[a*]

Samantha R. Lee, MS, MA[a]

Yara A. Halasa-Rappel, DMD, PhD[a]

Carlos Willian Rincon Perez, MS[b]

Arturo Harker Roa, PhD[b]

[a]Heller School for Social Policy and Management, Brandeis University

Waltham, Massachusetts 02454-9110, USA

[b]School of Government, University of Los Andes, Bogotá, Colombia

*Corresponding author. Email: shepard@brandeis.edu

Supporting Information S1 Table. Input data for target cities

Rank	Municipality[a]	Average notified release area dengue cases	Population 2021 DANE projection (total municipality)[c]	Area (total municipality, km^2)	Annual cost of vector control in full city[b]	% of the population in release areas	% of km^2 in release areas	Cost of *Wolbachia* deployment
1	Cali	8,018	2,264,748	562	$173,144	97.9%	16.5%	$8,973,571
2	Ibagué	2,999	542,724	1,377	$77,476	92.8%	1.9%	$2,269,484
3	Villavicencio	2,947	549,922	1,286	$101,230	92.0%	2.2%	$2,506,072
4	Cúcuta	2,824	787,891	1,132	$373,402	96.4%	4.4%	$4,363,719
5	Bucaramanga	2,767	614,269	153	$245,927	98.4%	14.8%	$1,989,085
6	Neiva	2,040	367,400	1,269	$51,381	93.4%	1.7%	$1,857,647
7	Barranquilla	1,744	1,297,082	154	$68,153	100.0%	42.9%	$5,783,242
8	Valledupar	1,142	544,134	4,185	$101,230	87.8%	0.6%	$2,234,434
9	Armenia	1,189	308,463	122	$38,484	97.5%	11.7%	$1,253,036
10	Pereira	946	480,803	608	$45,738	84.1%	2.9%	$1,524,673
11	Cartagena	713	1,043,926	595	$374,339	88.8%	7.4%	$3,864,257
**All**		**27,329**	**8,801,362**	**11,443**	**$1,650,504**	**94.8%**	**3.6%**	**$36,619,221**

[a]Cities are ranked in decreasing number of average annual dengue cases from 2010 through 2019, providing an ordering of cities according to their historical dengue burden; [b]Costs are in 2020 USD. [c]DANE= *Departamento Administrativo Nacional de Estadístic*. Complete citations are in the references section of the main manuscript.
