# Supplementary material for: Economic evaluation of Wolbachia deployment in Colombia: A modeling study
Source: PLoS One. 2025 Apr 30;20(4):e0307045. doi: 10.1371/journal.pone.0307045 (PMC12043165; doi:10.1371/journal.pone.0307045)
Supplement: S2 Table — (PDF) [file pone.0307045.s002.pdf]

# Supporting Information S2 Table

# Macro-costing approach to estimate the average cost of an outpatient visit and hospitalization (monetary amounts in 2020 USD)

For

Economic evaluation of *Wolbachia* deployment in Colombia: A modeling study

*Plos One*, 2025. https://doi.org/10.1371/journal.pone.0307045

By

Donald S. Shepard, PhD[a*]

Samantha R. Lee, MS, MA[a]

Yara A. Halasa-Rappel, DMD, PhD[a]

Carlos Willian Rincon Perez, MS[b]

Arturo Harker Roa, PhD[b]

[a]Heller School for Social Policy and Management, Brandeis University

Waltham, Massachusetts 02454-9110, USA

[b]School of Government, University of Los Andes, Bogotá, Colombia

*Corresponding author.  Email: shepard@brandeis.edu

**Supporting Information S2 Table. Macro-costing approach to estimate the average cost of an outpatient visit and hospitalization (monetary amounts in 2020 USD)**

| Item | Description | Formula | Parameters | Citation & Notes |
|---|---|---|---|---|
| **[1]** | The population of Colombia in 2018 | | 49,661,048 | Worldometer[36] |
| **[2]** | Consultations per person (adjusted based on undercounting for hospitalization) | | 4.62 | a |
| **[3]** | Bed-day equivalent factor for consultations | | 0.32 | Shepard[35] |
| **[4]** | Bed-day equivalents of average consultations | [2] × [3] | 1.4776 | |
| **[5]** | Hospitalizations derived from RIPS data | | 0.0787 | b |
| **[6]** | Share of all hospitalizations for all causes counted in RIPS | | 50.0% | OECD[34] |
| **[7]** | Estimated mean length of stay | | 3.00 | c |
| **[8]** | Hospital days (adjusted for RIPs undercount with 3 days LOS) | [7]×[9]/(1-[6]) | 0.4716 | |
| **[9]** | Total bed day equivalents | [4]+[8] | 1.9492 | |
| **[10]** | SGSSS amount for 2019 | | $249.04 | Ministerio[33] |
| **[11]** | Amount per bed day equivalent | [10]/[9] | $127.77 | |
| **[12]** | Amount per visit | [3] × [11] | $40.89 | |
| **[13]** | Amount per average hospitalization | [7] × [11] | $383.30 | |

Notes: [a] Derived from RIPS data (average number of consultancies per person); [b] Derived from RIPS data; [c] Derived from Police claims data and Gobierno de Colombia 2018.[32] LOS=length of stay; RIPS= *Registro Individual de Prestación de Servicios de Salud Municipio*; SGSSS= *Sistema General de Seguridad Social en Salud* [General System of Social Security in Health]; USD=United States dollars. Complete citations are in the references section of the main manuscript.
