# Supplementary material for: Economic evaluation of Wolbachia deployment in Colombia: A modeling study
Source: PLoS One. 2025 Apr 30;20(4):e0307045. doi: 10.1371/journal.pone.0307045 (PMC12043165; doi:10.1371/journal.pone.0307045)
Supplement: S3 Table — (PDF) [file pone.0307045.s003.pdf]

Supporting Information S3 Table

Health care cost of dengue cases by type of dengue diagnosis and setting using macro-costing (amounts in 2020 USD)

For

Economic evaluation of *Wolbachia* deployment in Colombia: A modeling study

Plos One, 2025. https://doi.org/10.1371/journal.pone.0307045

By

Donald S. Shepard, PhD[a*]

Samantha R. Lee, MS, MA[a]

Yara A. Halasa-Rappel, DMD, PhD[a]

Carlos Willian Rincon Perez, MS[b]

Arturo Harker Roa, PhD[b]

[a]Heller School for Social Policy and Management, Brandeis University

Waltham, Massachusetts 02454-9110, USA

[b]School of Government, University of Los Andes, Bogotá, Colombia

*Corresponding author. Email: shepard@brandeis.edu

Supporting Information S3 Table. Health care cost of dengue cases by type of dengue diagnosis and setting using macro-costing (amounts in 2020 USD)

Item	Description	**Formula**	**Severe dengue**	**Non-severe dengue[a]**	**Non-medical**	**Sources**
**[1]**	Cost of a hospitalization episode based on macro costing with RIPS volume, USD		$383	$383	$383	Macro costing[a]
**[2]**	The proportion of dengue patients hospitalized, based on RIPS data		67%	31%	0%	RIPS data
**[3]**	Cost of hospitalization per patient with any dengue, USD	[1] × [2]	$259	$117	$0	
**[4]**	The average number of ambulatory visits per dengue patient		3.14	1.91	0.00	RIPS data
**[5]**	Cost of an ambulatory visit based on macro costing with RIPS volume, USD		$40.89	$40.89	$40.89	Macro costing[a]
**[6]**	Average cost of ambulatory visits by dengue type, USD	[3] × [4]	$128.55	$78.25	$0.00	Authors' calculation
**[7]**	Cost of care received outside the medical system, USD		$0.00	$0.00	$1.50	Expert panel
**All**	**Estimated average total cost per dengue case (macro costing), USD**	**[3]+[6]+[7]**	**$387.18**	**$195.72**	**$1.50**	**Authors' calculation**

[a] Includes non-severe dengue with and without warning signs; [a] OECD, 2021.[34] Notes: RIPS= *Registro Individual de Prestación de Servicios de Salud Municipio*; USD=United States dollars. Complete citations are in the references section of the main manuscript.
