# Supplementary material for: Economic evaluation of Wolbachia deployment in Colombia: A modeling study
Source: PLoS One. 2025 Apr 30;20(4):e0307045. doi: 10.1371/journal.pone.0307045 (PMC12043165; doi:10.1371/journal.pone.0307045)
Supplement: S4 Table — (PDF) [file pone.0307045.s004.pdf]

Supporting Information S4 Table

Cost of dengue case by type based on SOAT tariff schedule and macro-costing (2019-20 US$)

For

Economic evaluation of *Wolbachia* deployment in Colombia: A modeling study

Plos One, 2025. https://doi.org/10.1371/journal.pone.0307045

By

Donald S. Shepard, PhD[a*]

Samantha R. Lee, MS, MA[a]

Yara A. Halasa-Rappel, DMD, PhD[a]

Carlos Willian Rincon Perez, MS[b]

Arturo Harker Roa, PhD[b]

[a]Heller School for Social Policy and Management, Brandeis University

Waltham, Massachusetts 02454-9110, USA

[b]School of Government, University of Los Andes, Bogotá, Colombia

*Corresponding author. Email: shepard@brandeis.edu

Supporting Information S4 Table. Cost of dengue case by type based on SOAT tariff schedule and macro-costing (2019-20 US$)[a]

Description	Severe dengue (medical)	Non-severe dengue (medical)	Non-medical	Row total
Share of dengue cases				
(1) Correctly reported	1.87%	27.13%	0.00%	29.00%
(2) Not reported	0.00%	11.00%	0.00%	11.00%
(3) Misdiagnosed	0.00%	20.00%	0.00%	20.00%
(4) Treated outside the medical system	0.00%	0.00%	40.00%	40.00%
(5) Column total	1.87%	58.13%	40.00%	100.00%
Derivation of overall cost per case				
(6) Cost per case by severity, from SOAT	$406.37	$188.02	$0.00	n.a.
(7) Share of correctly reported cases, from (1)	6.45%	93.55%	0.00%	100.00%
(8) Overall cost of cases treated in the medical sector (6) × (7)	$26.23	$175.89	$0.00	$202.11
(9) Overall health system cost of cases treated in the medical and non-medical sectors from SOAT, (5) × (6)	$7.61	$109.29	$0.00	$116.90
(10) Health system cost per case by severity, from macro-costing (Supporting Information S4 Table)	$387.18	$195.72	$0.00	n.a.
(9) Overall health system cost of cases treated in the medical and non-medical sectors from macro-costing (5) × (10)	$7.25	$113.77	$0.00	$121.02

[a] Notes: Non-severe cases include those both with and without warning signs. The shares of dengue cases are based primarily on results of the expert panel. In addition, correctly reported cases were allocated between severe and non-severe cases based on numbers in SIVIGILA [National Public Health Surveillance System]. Non-medical cases also incur out-of-pocket medication expenses of $1.50 and other household expenditures of US$22.86 per case. SOAT denotes *Seguro Obligatorio para Accidentes de Tránsito*, an obligatory insurance system from the payment schedule (tariffs) serve as reference prices; n.a. denotes not applicable.
