# Supplementary material for: Economic evaluation of Wolbachia deployment in Colombia: A modeling study
Source: PLoS One. 2025 Apr 30;20(4):e0307045. doi: 10.1371/journal.pone.0307045 (PMC12043165; doi:10.1371/journal.pone.0307045)
Supplement: S1 Text — (PDF) [file pone.0307045.s005.pdf]

Supporting Information S1 Text

Macro-costing approach

For

Economic evaluation of *Wolbachia* deployment in Colombia: A modeling study

Plos One, 2025. https://doi.org/10.1371/journal.pone.0307045

By

Donald S. Shepard, PhD[a*]

Samantha R. Lee, MS, MA[a]

Yara A. Halasa-Rappel, DMD, PhD[a]

Carlos Willian Rincon Perez, MS[b]

Arturo Harker Roa, PhD[b]

[a]Heller School for Social Policy and Management, Brandeis University

Waltham, Massachusetts 02454-9110, USA

[b]School of Government, University of Los Andes, Bogotá, Colombia

*Corresponding author. Email: shepard@brandeis.edu

Supporting Information S1 Text. Macro-costing approach

We adjusted the number of hospitalizations for underreporting in RIPS data. That adjustment compared the aggregate number of hospitalizations in RIPS data against the estimated number based on national hospital bed capacity using data from The Organization for Economic Cooperation and Development (OECD), hospital occupancy, and length of stay. RIPS reported 3,906,350 hospitalizations in 2018 (Supporting Information S2 Table). Our derived number was 7,806,386 based on a 3-day overall length of stay and an annual average of 64,162 occupied beds. The ratio of RIPS-reported to separately-projected numbers was 50% (i.e., 3,906,350 / 7,806,386). Using the macro-costing approach, a top-down costing approach that combines national statistics such as health care expenditure, the average length of hospital stays, and insurance coverage with a conversion value to estimate the average cost of hospitalization and outpatient visit.

Under the macro-costing approach, we then combined this information with dengue-specific utilization derived from Social Protection Ministry data, RIPS, and expert opinion to estimate the weighted average for a typical dengue case. We derived the proportion of dengue patients hospitalized and the average number of ambulatory services from RIPS data by type of dengue (see Supporting Information S3 Table). We estimated the cost of care in the non-medical sector based on the expert panel's assessment of the care needed assuming that these cases are mild classic dengue. Using macro-costing, to derive the cost of an average dengue case in Colombia, we combined the cost data with dengue epidemiological data. The epidemiological data, based on our panel of epidemiologic experts, estimated that 2% of dengue cases were severe dengue, 58% were non-severe dengue (including those with and without warning signs) treated in the medical sector, and 40% were dengue cases treated in the non-medical sector.
