# Supplementary material for: Economic evaluation of Wolbachia deployment in Colombia: A modeling study
Source: PLoS One. 2025 Apr 30;20(4):e0307045. doi: 10.1371/journal.pone.0307045 (PMC12043165; doi:10.1371/journal.pone.0307045)
Supplement: S2 Text — (PDF) [file pone.0307045.s006.pdf]

Supporting Information S2 Text

Exploratory approach to costing by health system tier

For

Economic evaluation of *Wolbachia* deployment in Colombia: A modeling study

Plos One, 2025. https://doi.org/10.1371/journal.pone.0307045

By

Donald S. Shepard, PhD[a*]

Samantha R. Lee, MS, MA[a]

Yara A. Halasa-Rappel, DMD, PhD[a]

Carlos Willian Rincon Perez, MS[b]

Arturo Harker Roa, PhD[b]

[a]Heller School for Social Policy and Management, Brandeis University

Waltham, Massachusetts 02454-9110, USA

[b]School of Government, University of Los Andes, Bogotá, Colombia

*Corresponding author. Email: shepard@brandeis.edu

Supporting Information S2 Text: Exploratory approach to costing by health system tier

Researchers are aware that the accuracy of each field in an administrative database, such as RIPS, depends on the precision with which sites and administrators enter and subsequently check and clean that item. The accuracy can vary between fields. We explored using the RIPS data to classify dengue cases by what we called the "tier," the most intensive setting in which a patient received services during a calendar. These were: hospital, emergency, consultations, and procedures. The resulting data, however, were not consistent with the epidemiological literature about dengue in the region. Our attempted breakdowns by tier in RIPS showed only a small number of patients with a dengue hospitalization, but each person with a hospitalized case was reported to have had three dengue hospitalizations. As hospitalization for dengue in a year is relatively rare, having two in a year should be very rare and three extremely rare. For this reason, we concluded that the necessary fields in the RIPS data were not sufficiently accurate to allow subdividing RIPS utilization and costs by setting or "tier."
