# Supplementary material for: Economic evaluation of Wolbachia deployment in Colombia: A modeling study
Source: PLoS One. 2025 Apr 30;20(4):e0307045. doi: 10.1371/journal.pone.0307045 (PMC12043165; doi:10.1371/journal.pone.0307045)
Supplement: Attachment — Submitted filename: Pone-response04.docx [file pone.0307045.s007.docx]

Response to reviewer regarding an earlier submission (PONE-D-23-41408). Note: For clarity we have numbered the comments and our responses.

1	Comment from Reviewer #1: This paper presents an economic evaluation of Wolbachia deployment in Colombia. This an interesting analysis and the conclusions are clear and well balanced.

1	Response: We appreciate this endorsement.

2	Comment. My major comment is that I found certain parts of the methodology unclear. I’m unclear how the difference in DALY weights and cost of illness between outpatient and hospitalised cases were accounted for in cases that were treated in the medical sector

2	Response. We cite the paper by Zeng et al (2018), our reference 23, which we believe provides the most comprehensive analysis of the dengue burden per case. It notes that for overall burden, mortality, although rare, contributes substantially to the DALY burden. Among non-fatal cases, the persistent or long term component is also an important component. Our major source of dengue data by city, the surveillance data, did not separate hospitalized and ambulatory cases. Also, our adjustment factors for unreported and mis-diagnosed cases do not distinguish between ambulatory and hospitalized cases. For all these reasons, we used an overall estimate of the average burden per case (combining hospitalized, ambulatory, and non-medical cases).

3	Comment. More information is needed in the methods regarding the different perspectives used and how the indirect costs (productivity costs) were calculated.

3	Response. We expanded the discussion and sources of indirect costs, including a recent (2023) publication that provided a new empirical estimate of Colombia’s threshold value. Our discussion with knowledgeable official indicates that the SOAT tariffs appear to represent a reasonable long term average.

4	Comment. I found it unclear why (and how) mandatory road traffic tariffs being used for the cost of dengue cases. In the discussion, a macro-costing approach is mentioned but I could not see this in the methods.

4	Response. We explain how the SOAT tariffs serve as a set of reference tariffs and, on average seem to reflect average compensation.

5	Comment. In addition: “A positive ICER below Colombia’s per capita GDP ($5,312) indicates that the intervention is highly cost-effective.” I would disagree that this no longer valid. The 1*times per capita GDP threshold for being “highly cost-effective” has been widely criticised and no longer endorsed by the WHO.

5	Response. We appreciate this important comment. As a result, we reviewed the literature on cost-effectiveness thresholds in low and middle-income countries and revised our analysis of thresholds. We now acknowledge that WHO no longer recommends the 1x and 3x multiples of per capita GDP. As a more appropriate method, we have selected the approach of Pichon-Riviere et al (2023), our reference 40, and updated the calculation of indirect costs and thresholds for cost-effectiveness accordingly.

6	Comment. In Table 1 you presented Annualization factors. On what costs were these applied – I assume the Cost of Wolbachia deployments but this should be clearly mentioned in the in methods.

6	Response. We previously used the annualization factors to derive the present value factor (with a value of 8.53 for 10 years). However, the current revision calculates the present value factor directly (as described in the methods) and applies it to generate the present value of treatment costs and indirect costs. As the *Wolbachia* costs were incurred over just two years, we discounted the second year directly and did not need the annualization factor

7	Minor comments
Table 1: “DALY/case (mortality [33%] and morbidity [67%])” – a little unclear. I would add the breakdown as a footnote of the table

7	Response. We removed the parenthetical expression from the label of DALY/case. We started to add the breakdown to the footnote, as suggested. However, in re-reading the manuscript we felt that the explanation would fit better in the text where we discuss DALYs per case and added it there.

8	Comment. Table 1 : legend Obligatorio para Accidentes de Tránsito] Compulsory Insurance for Traffic Accidents]; USD=United States dollars. Monetary amounts in 2020 USD (or the closest year available) at market exchange rates. 9 Sources: P15 from Excel (=pmt(3%,10,-1); P16 = 1 / P15; P17 from the World Bank,18 and all others from authors’ calculations. Apologies to be an error with the squire brackets

8	Response. We re-wrote the note to Table 1 to clarify it.
