# Supplementary material for: Economic evaluation of Wolbachia deployment in Colombia: A modeling study
Source: PLoS One. 2025 Apr 30;20(4):e0307045. doi: 10.1371/journal.pone.0307045 (PMC12043165; doi:10.1371/journal.pone.0307045)
Supplement: Attachment — Submitted filename: Col Econ_Comments_Feb2025h.docx [file pone.0307045.s009.docx]

**Response to reviewer**

We thank the reviewer for the constructive comments. In the course of responding to these comments, we also incorporated some editorial refinements.

Reviewer 3, Lines 59-61: Please include the number of DENV cases during the specified years and the associated costs. This information is fundamental from an economic perspective.

Response: We have expanded and renumbered the tables to incorporate this. The new Table 2 has two estimates of the number of dengue cases. In column 4 we report the average notified release area dengue cases. In column 5 we report the projected release area dengue cases without treatment. The latter number includes adjustments for under-reporting. In column 3 of the new Table 3 we report the current cost of dengue cases (without the *Wolbachia* intervention).

Reviewer 3, Lines 72-74: It is crucial to clarify which *Wolbachia* strain was used in Australia and to describe the environmental conditions of Cairns. Additionally, clarify whether the same *Wolbachia* strain will be used in Colombia.

Response. We expanded the phrase around Cairns, Australia, adding “using *Ae. aegypti* infected with the *w*Mel strain of *Wolbachia"* (see line 67). Under the replacement strategy, we revised the sentence to read ” Singapore releases only male *Wolbachia* infected mosquitoes (wAlbB strain)." (see line 72). We modified the sentence about the Yogyakarta to read “found that the *Wolbachia* (*w*Mel strain) replacement strategy reduced all virologically-confirmed symptomatic dengue cases…” (line 79). We modified the sentence about Niteroi, Brazil to read “*w*Mel-*Wolbachia* reduced the incidence of dengue by 69%,..." (line 87). We modified the background on Colombia to read "In Colombia, pilot *w*Mel-*Wolbachia* releases began in the city of Bello..." (line 92).

Reviewer 3, General: Ensure that all scientific names, such as *Wolbachia*, are italicized consistently throughout the manuscript.

Response. We have checked the manuscript and capitalized as necessary.

Reviewer 3, Lines 140-141: Please specify the source of this information.

Response. We modified Table 1 to change the column heading to “Description and source” and added the source for each item.

Reviewer 3, Lines 142-144: The authors should consider that the CRT conducted in Yogyakarta has different socio-economic implications when extrapolated to Colombia. This factor must be adjusted based on the analysis.

Response. We addressed this concern in the discussion in the paragraph beginning “As a *Wolbachia* program depends….” (line 517)

Reviewer 3, Line 160:"P1" should be corrected (P!).

Response. Done

Reviewer 3, Line 229: The statement that the *Wolbachia* program in Colombia will result in a 75% reduction in dengue cases appears to be based on Yogyakarta's results. If this percentage is used as a fixed estimate, it could pose a risk in the coming years. It is recommended to present a range instead of a fixed percentage.

Response. To address this important question, we added sentences in the discussion beginning “Our projected 75% efficacy is likely conservative…” (line 532)

Reviewer 3, Lines 309-312: Among the listed cost categories, which are the most expensive? The authors should propose or estimate strategies to reduce costs, potentially through intersectoral funding, including contributions from private companies.

Response. We added the sentences beginning “In Cali, the most expensive…” (line 488)

Reviewer Table 2: What is the budget allocated by each city for vector control? This information is crucial for determining the feasibility of the PR implementation.

Response: Table 3, Column 4 shows this current cost. We added a comment to the discussion beginning with “While we project reductions in conventional vector control….” (line 485)

Reviewer 3, Discussion. The manuscript should discuss how each city (municipality) will formalize agreements and incorporate the PR strategy as part of the national dengue control strategy. Additionally, consider how private companies could contribute to these efforts.

Response. We added the sentences beginning: “If a city wished to implement a *Wolbachia* program, a portfolio of financing approaches….” (line 567)

Reviewer 3, Lines 532-534: It is important to incorporate the most common risks associated with costs into the model. Other potential risks, such as pandemics, changes in government, and budget reductions, should also be considered.

Response: We added a paragraph to discuss risks and resilience of the *Wolbachia* approach beginning “Any public health program risks interference….” (line 514).

Reviewer 3, Lines 568-574: The authors could propose a hybrid model combining vaccines and traditional vector control measures to reduce implementation costs. This approach could facilitate cost-sharing with other institutions and enhance the accessibility and affordability of the PR strategy.

Response: We responded insofar as our data allowed by revising our conclusion with the sentence beginning “*Wolbachia* is likely to be the more cost-effective or cost-saving …” (line 605). More specific recommendations would require the future development and calibration of more detailed epidemiologic and economic models.

Reviewer 3, Conclusion, Lines 610-613: Municipalities generally have very limited budgets (especially in LATAM), and mobilizing resources requires scaling up efforts to the national level. A gradual integration of the *Wolbachia* strategy within a holistic integrated vector control framework is necessary. Otherwise, a standalone strategy may be financially unfeasible.

Response: We added a sentence to the discussion beginning “To become cost-effective, this community-based dengue control…” (line 540) We believe that previously mentioned revisions also address this comment. See the sentences on financing “…a portfolio of financing approaches deserve consideration….” (lines 567) and the revision to the conclusions “Wolbachia is likely to be the more cost-effective or cost-saving …” (line 605)
